# Establishment of a Stable *Anopheles melas* Laboratory Colony for the First Time and Characterization of Its Pyrethroid Resistance Profile in Senegal

**DOI:** 10.3390/insects17080763

**Published:** 2026-07-25

**Authors:** Mohamed Abderemane Nourdine, Pape Cheikh Sarr, Abdoulaye Niang, Assane Ndiaye, Lina Ibrahim Abdallah, Idrissa Yèro Sarr, Lassana Konaté, Ousmane Faye, Oumar Gaye, Ellen M. Dotson, El Hadji Amadou Niang, Ousmane Sy

**Affiliations:** 1 Laboratoire d’Écologie Vectorielle et Parasitaire, Département de Biologie Animale, Université Cheikh Anta Diop, Dakar BP 5005, Senegal; akhysarr@gmail.com (P.C.S.); abdoulaye20.niang@ucad.edu.sn (A.N.); ndiayeassane8888@gmail.com (A.N.); lina.ibrahimabdallah@ucad.edu.sn (L.I.A.); idrissayero.sarr@ucad.edu.sn (I.Y.S.); lassanaknt33@gmail.com (L.K.); jogomaye@yahoo.fr (O.F.); elhadjiamadou.niang@ucad.edu.sn (E.H.A.N.); 2 Malaria Reasearch Capacity Development in West and Central Africa (MARCAD-Plus Consortium), Faculté de Médecine, Pharmacie et D’Odontologie, Université Cheikh Anta Diop, Dakar BP 5005, Senegal; oumarg613@gmail.com; 3 Entomology Branch, Division of Parasitic Diseases and Malaria, Centers for Disease Control and Prevention (CDC), Atlanta, GA 30329, USA; ebd6@cdc.gov

**Keywords:** *Anopheles melas*, laboratory colonization, pyrethroid resistance, piperonyl butoxide (PBO), residual transmission, Senegal

## Abstract

In Senegal, scaling up vector control has significantly reduced malaria, but coastal transmission remains a challenge due to secondary vectors like *Anopheles melas*. This exophilic and zoophilic species evades conventional indoor interventions, yet its bionomics and insecticide resistance profiles remain poorly characterized due to difficulties in laboratory colonization. *Anopheles melas* larvae were collected from brackish breeding sites in Mbine Coly (Mbour District). A laboratory colony was established from 26 PCR-confirmed founder females and stabilized up to the F17 generation by optimizing water salinity (10 g/L) and transitioning to communal oviposition. Standardized WHO tube bioassays were conducted on generations F2 to F9 to evaluate susceptibility to pyrethroids, organophosphates, and carbamates, alongside synergist tests using 4% piperonyl butoxide (PBO). The colony showed confirmed resistance to alpha-cypermethrin (81.7% mortality) and suspected resistance to deltamethrin (91.3%) and permethrin (94.3%). Pre-exposure to PBO fully restored susceptibility to deltamethrin and permethrin (100%), and significantly increased alpha-cypermethrin mortality to 96.3% (*p* < 0.001). While metabolic resistance typically involves multiple enzyme families including cytochrome P450s, glutathione S-transferases (GSTs), and esterases, these synergist results demonstrate a predominant role of cytochrome P450-mediated detoxification pathways in this population. Moderate-intensity resistance was confirmed for alpha-cypermethrin at 5× diagnostic doses. Conversely, the population was 100% susceptible to pirimiphos-methyl and bendiocarb. This study reports the successful long-term colonization of a wild *An. melas* strain for the first time and provides the first comprehensive evidence of multi-insecticide resistance in this population. The presence of high-intensity, metabolic pyrethroid resistance strongly justifies the immediate deployment of next-generation PBO-LLINs and the integration of organophosphates or carbamates in indoor residual spraying rotations to manage resistance in coastal Senegal.

## Simple Summary

Malaria remains a major health problem in Senegal. Traditional control tools like standard bed nets target mosquitoes that bite indoors, but some mosquito species bite outdoors and survive in salty coastal waters, escaping these measures. This study aimed to successfully breed one such coastal mosquito species in a laboratory and test its vulnerability to different public health chemicals. The researchers successfully established a stable laboratory colony by finding the perfect water saltiness for their survival. Tests revealed that these mosquitoes have developed a strong resistance to standard chemicals used on common bed nets. However, the researchers discovered that adding a specific neutralizing chemical completely blocks this resistance, making the nets effective again. Furthermore, the insects remained entirely vulnerable to two other classes of public health chemicals. These findings are highly valuable for society because they show health authorities exactly which tools to deploy. Specifically, the study recommends distributing new, advanced bed nets and rotating chemicals used in home spraying to protect coastal communities and help eliminate malaria.

## 1. Introduction

Senegal has scaled up insecticide-based vector control interventions, notably Indoor Residual Spraying (IRS) and Long-Lasting Insecticidal Nets (LLINs), over the past two decades. Despite steady progress, the country recorded 232,465 confirmed cases and 199 deaths in 2023, illustrating the persistence of malaria endemicity with marked regional disparities [1]. While incidence has significantly declined in the North and Central regions, offering a genuine opportunity to achieve the 2030 elimination goal set by health authorities, controlling transmission remains complex in transition zones. Previous studies have reported the presence of *Anopheles melas* in several coastal and estuarine areas, where it plays a central role in malaria dynamics [2,3,4]. Within the *Anopheles gambiae* sensu lato complex, *Anopheles melas* stands out as a highly specialized coastal vector. Unlike its fresh-water sibling species, such as *An. gambiae* sensu stricto, *An. arabiensis* and *An. coluzzii*, which breed in temporary rain-fed pools, *An. melas* is strictly halophilic. Its larvae develop in brackish water environments, including coastal mangroves, lagoons, and salt marshes, adapted to high salinity through advanced physiological osmoregulatory functions. However, the epidemiological, ecological, and entomological factors enabling *An. melas* to maintain residual transmission are not yet fully characterized.

In West-Central Senegal, this species ensures transmission alongside *An. arabiensis*, whose populations already exhibit high resistance to pyrethroids [5]. To address this threat, the National Malaria Control Programme (NMCP) introduced new strategies in its 2021–2025 National Strategic Plan, including the deployment of next-generation piperonyl butoxide (PBO) nets. However, shifts in species complex composition, with an increasing contribution from secondary vectors such as *An. melas*, pose a major challenge. Its zoophilic and exophilic behaviour places it beyond the reach of conventional interventions targeting endophilic vectors [6]. We hypothesize that *An. melas* could become the predominant local species by gradually replacing *An. arabiensis*, thereby becoming the primary driver of residual malaria within its distribution range. It is therefore imperative to document the bionomics, distribution, and genetic structure of its populations. Understanding its larval bionomics and genetic diversity would allow for the anticipation of epidemiological shifts, the assessment of insecticide resistance allele spread, and the early detection of entomological risks in pre-elimination zones. In the face of rising resistance, characterizing the susceptibility of *Anopheles melas* is now crucial to adapt NMCP tools and ensure the effectiveness of future public health investments.

## 2. Materials and Methods

### 2.1. Study Area and Entomological Collection

**Figure 1:**
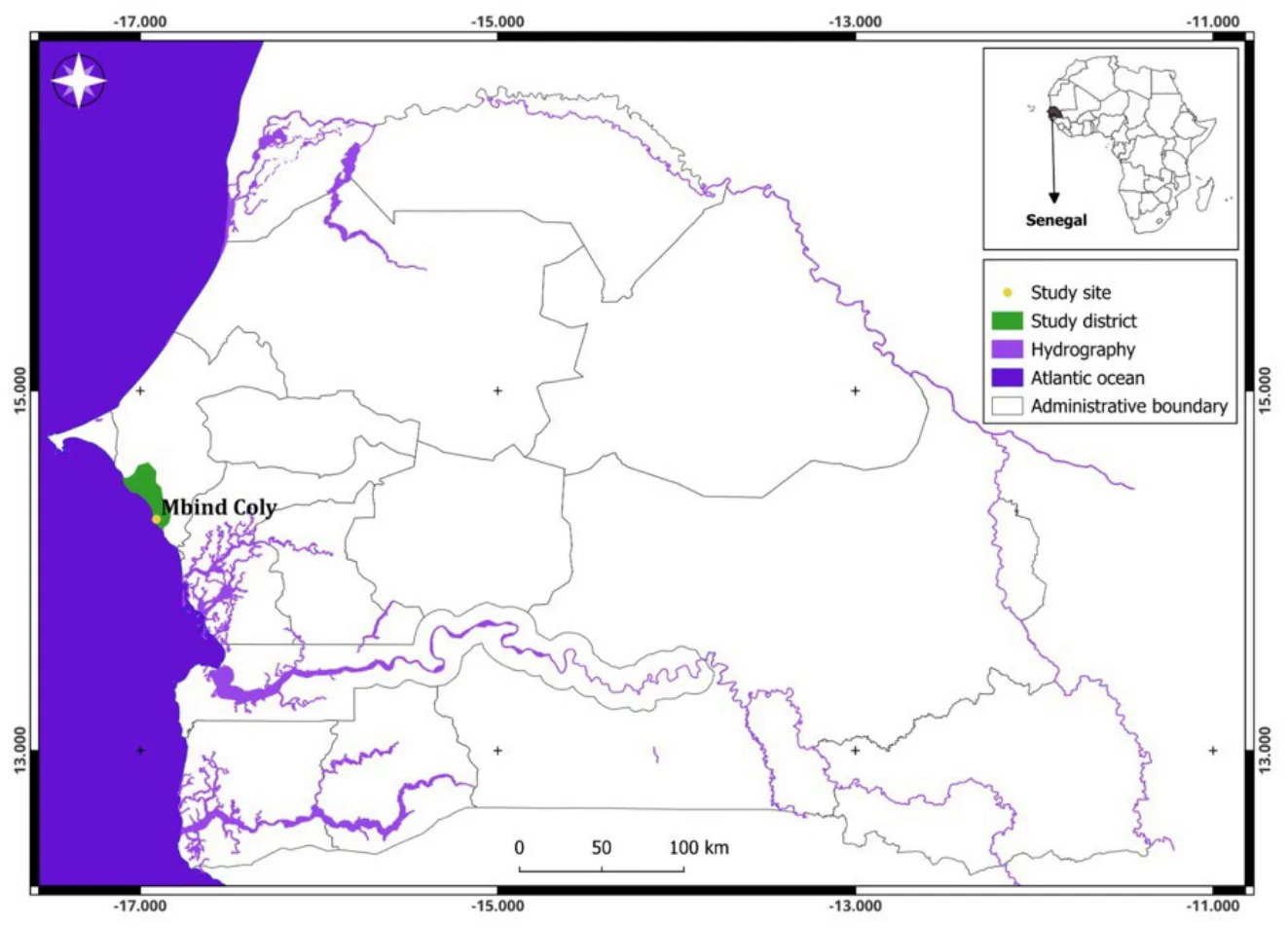
Geolocation of Mbine Coly village in the Mbour district.

Larval surveys were conducted on 26 September 2024, in Mbine Coly (14°17′10″ N, 16°54′30″ W), located in the coastal area of the Mbour District, western Senegal (Figure 1). The village is surrounded by extensive mangrove swamps and brackish flood zones, which create typical “crab hole”-type larval breeding sites. The area is characterized by a coastal Sudano-Sahelian climate, features a short rainy season from July to October and a prolonged dry season from November to June, with an annual rainfall averaging around 500–600 mm. Larvae were collected using the dipping method in waters with salinity levels ranging from 10 to 18 g/L.

### 2.2. Colonization and Laboratory Rearing Optimization

**Figure 2:**
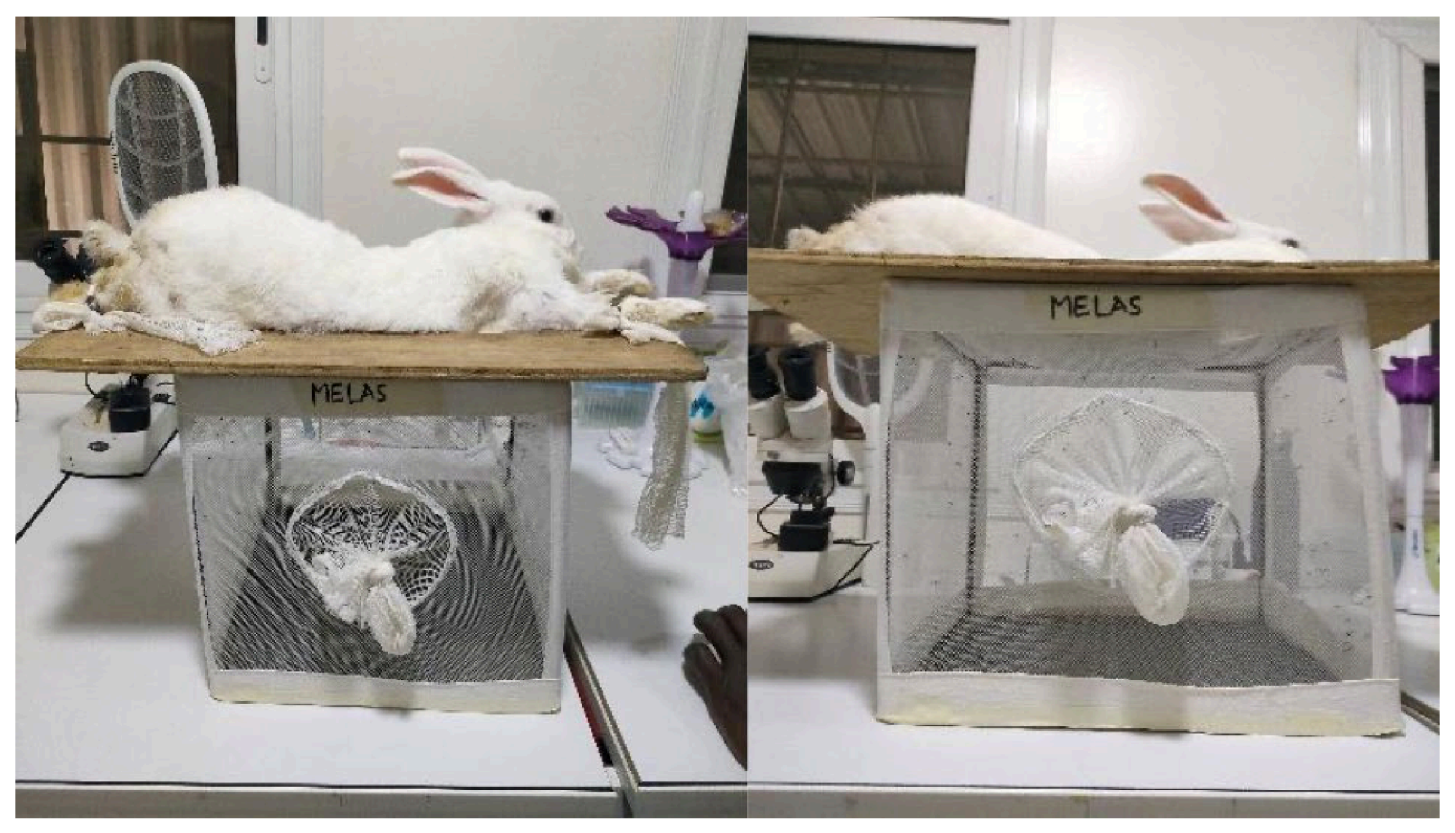
Blood-feeding of *Anopheles melas* females on a rabbit. The rabbits used for blood-feeding were obtained from the official animal facility of the Insectary at the Laboratory of Parasite and Vector Ecology (LEVP), Université Cheikh Anta Diop (UCAD), Dakar, Senegal.

The *An. melas* colony was established from 26 PCR-identified founder females (F0). Rearing was conducted under controlled insectarium conditions (25 °C and 70–80% humidity). A rigorous protocol was implemented to ensure the sustainability of the strain:

Blood-feeding and Oviposition: To stimulate egg production, F0 females were deprived of sugar solution for 48 h before being blood-fed on a rabbit. The rabbits were shaved on the ventral area to facilitate contact with mosquitoes, and the feeding process lasted approximately 30 min per cage (Figure 2). Humidity was maintained using damp cloths placed over the cages. Following the blood meal, females were isolated in Eppendorf tubes for individual forced oviposition on moist filter paper. Transition to communal oviposition for subsequent generations was stabilized using cups containing 1 cm of water, where mosquitoes laid eggs directly on the surface.

Salinity Optimization: Although the species showed survival in brackish water at 18 g/L, our tests demonstrated that 10 g/L is the optimal salinity. This concentration allows for faster hatching and larval growth compared to freshwater, while avoiding the decline in fecundity observed at 18 g/L.

Larval Monitoring Schedule: The maintenance cycle was standardized to 12 days to ensure homogeneous population sizes:

D1–D2: Flooding (100 mL) and initial hatching (24–48 h).

D3–D5: To standardize larval development and avoid overcrowding artifacts, newly hatched first-instar (L1) larvae were carefully quantified and allocated to plastic rearing trays at a fixed density of 400 larvae per 1.5 L of brackish water (10 g/L salinity). Larvae were maintained under a controlled nutritional regimen with a progressive feeding schedule, where the daily food ration was incrementally adjusted from 50 mg to 120 mg per tray according to the developmental stages.

D6–D8: Critical stage involving complete brackish water renewal via filtration to prevent fouling, and increased ration (up to 250 mg).

D9–D12: Daily pupae collection and gradual cessation of feeding.

To optimize rearing conditions, preliminary trials evaluated egg hatching and larval survival across three salinity concentrations (0, 10, and 18 g/L). Larval mortality and development times were monitored daily. Differences in survival rates were analyzed using a Chi-squared test in RStudio (version 4.3.1).

### 2.3. Species Identification

Identification was performed by PCR according to the protocol by Wilkins et al. (2006) [7] on 26 F0 females that had oviposited individually. This analysis confirmed that the study population consisted of 100% *Anopheles melas*.

### 2.4. Susceptibility Bioassays (WHO)

To ensure a homogeneous population for susceptibility testing, field-caught mosquitoes (F0) were maintained until the F9 generation, and tests were conducted on specimens from the F2 to F9 generations. A total of 1314 specimens (F2 to F9) were tested following standardized WHO guidelines [8,9]. Control groups (exposed to solvent only) were systematically included to validate each test series.

#### 2.4.1. Diagnostic and Intensity Tests (1×, 5×, 10×)

The WHO tube protocol was applied to evaluate susceptibility to pyrethroids (Deltamethrin 0.05%, Permethrin 0.75%, and Alpha-cypermethrin 0.05%), organophosphates (Pirimiphos-methyl 0.25%), and carbamates (Bendiocarb 0.1%) [9,10]. Groups of 20 to 25 mosquitoes per tube were exposed to impregnated papers for 60 min. Individuals were then transferred to holding tubes with access to a 10% sugar solution. Final mortality was recorded after a 24 h recovery period at 27 °C (±2 °C). For alpha-cypermethrin, 5× and 10× doses were used to characterize the intensity of resistance.

#### 2.4.2. PBO Synergist Tests

To detect the involvement of metabolic mechanisms (cytochrome P450s), pre-exposure tests to 4% Piperonyl Butoxide (PBO) were performed. Four groups were established:

Control: Neutral paper.

Synergist only: 4% PBO (60 min) to verify the absence of inherent toxicity.

Insecticide only: 1× diagnostic dose (60 min).

PBO + Insecticide: Pre-exposure to PBO (60 min) followed immediately by exposure to the 1× insecticide dose (60 min).

### 2.5. Data Analysis and Interpretation Criteria

Mortality was interpreted according to WHO thresholds: susceptible (98–100%), suspected resistance (90–97%), and confirmed resistance (<90%). If control mortality was between 5% and 20%, Abbott’s formula was applied to correct mortality rates. The involvement of metabolic mechanisms was confirmed if PBO pre-exposure restored susceptibility to a level ≥ 98%. As specific diagnostic and intensity doses for *Anopheles melas* are not explicitly defined in current WHO guidelines, the interpretive thresholds and intensity categories (<98% mortality at 5× and ≥98% mortality at 10× for moderate intensity) established for the *Anopheles gambiae* complex were applied.

### 2.6. Statistical Analyses

Observed mortality rates from bioassays were interpreted according to WHO criteria: full susceptibility (mortality ≥ 98%), suspected resistance (90–97%), and confirmed resistance (<90%). To validate the robustness of the results, the following analyses were performed:

Fisher’s exact test (or Pearson’s Chi-square test, *χ*2) was used to compare mortality rates between the insecticide alone (1×) and after pre-exposure to the synergist (PBO). To account for multiple comparisons across the tested insecticides and control the Type I error rate, *p*-values from Fisher’s exact tests were adjusted using the Benjamini–Hochberg False Discovery Rate (FDR) procedure [11]. Statistical significance was set at an adjusted *p*-value < 0.05. All statistical analyses were performed using R software (version 4.3.1).

## 3. Results

### 3.1. Species Identification and Establishment of the F1 Colony

**Figure 3:**
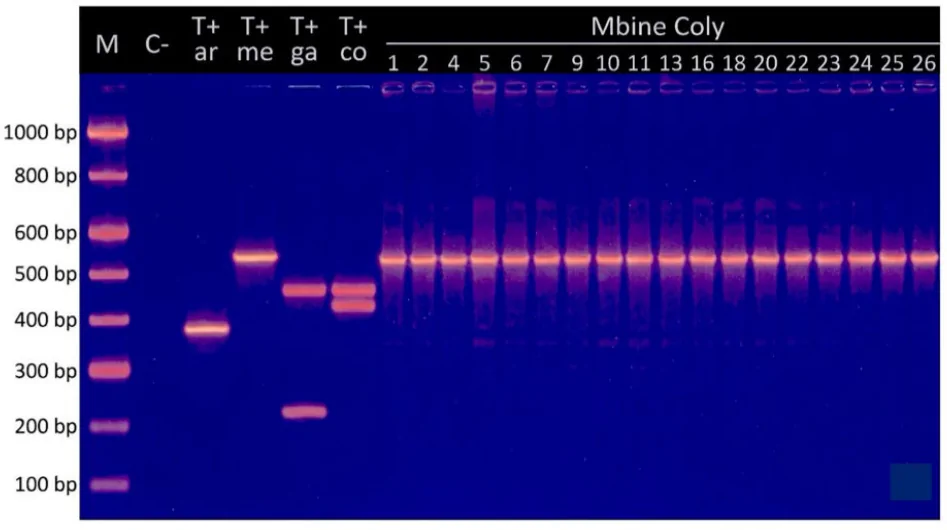
Agarose gel electrophoresis (2%) for the molecular identification of the *Anopheles gambiae* complex according to the Wilkins et al. (2006) protocol [7].

Molecular identification by PCR confirmed the exclusive presence of *Anopheles melas* (100%) among the 26 founder females collected in Mbine Coly. Electrophoretic analysis of the PCR products on a 2% agarose gel revealed a single, distinct diagnostic band at 528 base pairs (bp) for all successful samples, in strict accordance with the Wilkins et al. (2006) diagnostic framework [7]. No amplification products corresponding to freshwater sibling species (such as *An. gambiae s.s.* or *An. coluzzii*) were observed (Figure 3). Generations F2 to F9 were subsequently used for susceptibility bioassays.

Lane M: 100 bp DNA ladder; Lane C-: Negative control (H2O); Lane T+ ar: *An. arabiensis* positive control (387 bp); Lane T+ me: *An. melas* positive control (528 bp); Lane T+ ga: *An. gambiae* positive control showing the shared complex band at 463 bp and the species-specific fragment at 221 bp; Lane T+ co: *An. coluzzii* positive control showing the shared complex band at 463 bp and the species-specific fragment at 426 bp; Lanes 1–26: PCR amplification products from field-caught founder females from Mbine Coly, all displaying the single diagnostic band at 528 bp matching the *An. melas* control.

### 3.2. Colonization Success and Rearing Parameters

The colonization trial of the *An. melas* population from Mbine Coly yielded conclusive results, enabling the transition from a wild population to a stabilized laboratory strain. As detailed in Table 1, the life cycle duration was thoroughly monitored across generations; egg hatching required 24.2 ± 2.1 h at 10 g/L, and larval development to the pupal stage spanned 8.4 ± 0.8 days under standardized conditions. Furthermore, adult females exhibited a high feeding success rate on rabbits, with an average of approximately 80% of females becoming fully engorged within the 30 min feeding period, ensuring robust fecundity and high egg production for subsequent generations.

Developmental kinetics: Under controlled conditions (25 °C, 70–80% RH), the average egg-to-adult cycle duration was 10 to 12 days. The colony was successfully established by adjusting water salinity. Rearing larvae in 10 g/L saline water significantly optimized colonization success compared to freshwater (0 g/L) and higher salinity (18 g/L) (Table 1). Specifically, it accelerated egg hatching time to 24.2 ± 2.1 h and reduced cumulative larval mortality by 15.5% (χ^2^ = 6.42, *p* = 0.011).

Productivity and fecundity: Starting from the 26 founder females (F0), the oviposition rate increased steadily. The transition from forced oviposition (F1-F2 generations) to communal oviposition marked a turning point in the colony’s productivity, providing sufficient numbers (over 1000 individuals per generation) to conduct large-scale bioassays (1×, 5×, and 10× doses).

Strain stability: The colony maintained constant vigor from generation F2 to F9, the period during which resistance tests were performed. Currently, the strain is sustained at generation F17, confirming its full adaptation to insectarium conditions.

### 3.3. Pyrethroid Susceptibility and Synergist Tests (PBO)

**Figure 4:**
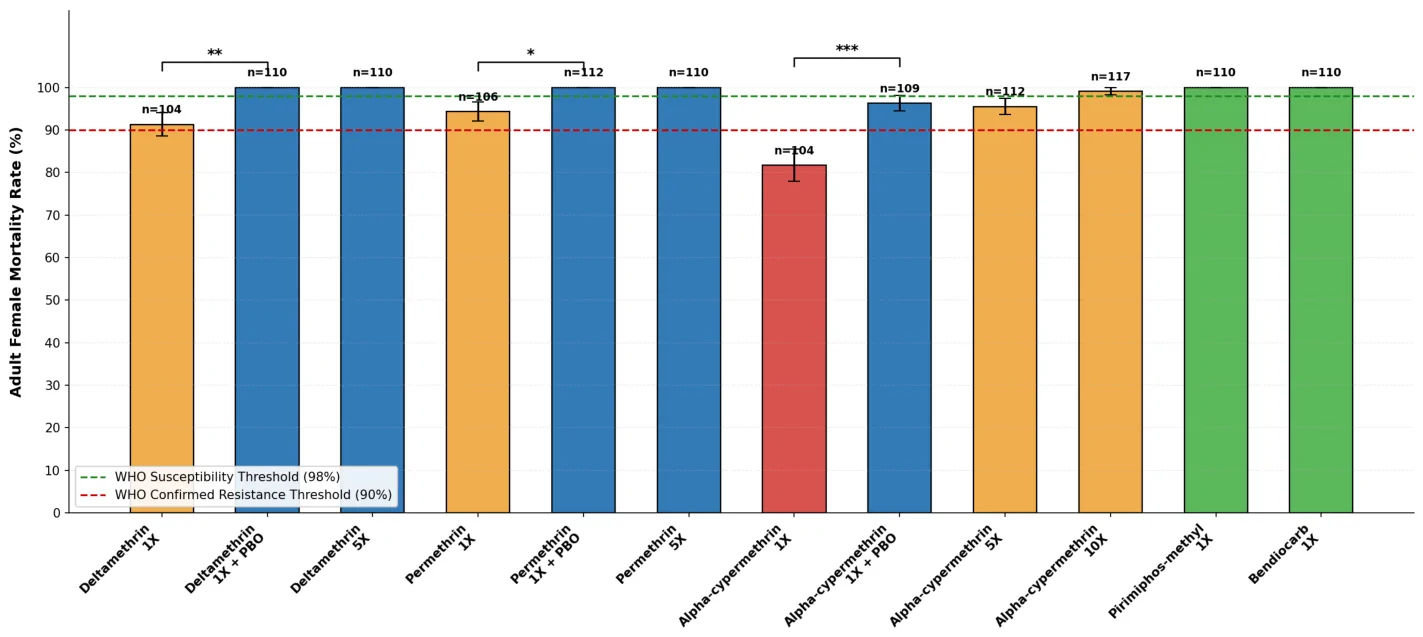
Insecticide susceptibility status, resistance intensity, and synergistic effect of piperonyl butoxide (PBO) against pyrethroids in the colonized *Anopheles melas* population. Note: Error bars represent 95% confidence intervals. Asterisks indicate statistically significant differences between insecticide alone and insecticide + PBO treatments based on Fisher’s exact test with Benjamini–Hochberg adjustment (* *p* < 0.05, ** *p* < 0.01, *** *p* < 0.001). n represents the total number of mosquitoes tested per condition.

In all experimental replicates, the control groups exposed to carrier-solvent papers exhibited strictly 0% mortality, confirming the validity of the bioassays and indicating that no Abbott’s mathematical correction was required for the treatment datasets. The bioassays revealed varying levels of resistance across molecules, as confirmed by significance analyses (Fisher’s exact test):

Deltamethrin and Permethrin: Suspected resistance was observed (91.3% and 94.3% mortality, respectively) (Figure 4). Pre-exposure to PBO led to full restoration of susceptibility (100%), representing a statistically significant increase (adjusted *p* = 0.006 for deltamethrin; adjusted *p* = 0.035 for permethrin).

Alpha-cypermethrin: Resistance was confirmed with 81.7% mortality. The addition of PBO increased this rate to 96.3%. This improvement was highly significant (adjusted *p* < 0.001), demonstrating the predominant role of cytochrome P450 enzymes in this population (Figure 4).

### 3.4. Resistance Intensity

Intensity bioassays (5× and 10×) further defined the strength of this resistance:

For deltamethrin and permethrin, full susceptibility was achieved at the 5× dose. For alpha-cypermethrin, mortality increased from 81.7% (1×) to 95.5% (5×) and reached 99.1% at the 10× dose. Following WHO’s evaluation criteria extrapolated from *An. gambiae* s.l., the persistence of survivors at 5× combined with a mortality rate ≥ 98% at 10× confirms a moderate-intensity resistance for this molecule (Table 2).

### 3.5. Susceptibility to Organophosphates and Carbamates

In contrast to pyrethroids, the Mbine Coly strain exhibited full susceptibility (100%) to pirimiphos-methyl and bendiocarb. The difference in mortality between these classes and alpha-cypermethrin is highly significant (*p* < 0.0001), validating the absence of cross-resistance and highlighting the potential for insecticide rotation strategies (Table 3).

## 4. Discussion

The management of residual malaria transmission in West Africa relies on a detailed understanding of secondary vectors such as *Anopheles melas.* Our results in Mbine Coly reveal that a salinity of 10 g/L is optimal for larval development. This observation corroborates previous studies on this species, which highlight its high plasticity (euryhalinity); however, high salinity levels close to seawater (~35 g/L) slow down larval growth [2,3,4,5,6,7,8,9,10,11,12]. The use of reconstituted brackish water at 10 g/L appears to offer an ideal compromise, avoiding osmotic stress while mimicking the conditions of mangrove areas or estuary margins encountered in Mbour. The transition from forced oviposition to communal oviposition starting from the F2 generation was the key factor in increasing population size. Maintaining the colony until F9 for susceptibility testing ensures the homogeneity of the tested samples. The successful colonization beyond F12, reaching F17, indicates genetic adaptation to insectarium conditions, making this Mbine Coly strain a valuable tool for future screening tests of new insecticidal molecules [13].

Maintaining a stable laboratory colony of *Anopheles melas* presents severe technical and biological challenges, which explains why successfully established strains of this species are extremely rare. The primary obstacle resides in its stenogamous mating behavior; unlike freshwater vectors, wild *An. melas* adults often fail to swarm and copulate naturally within standard laboratory mesh cages, frequently requiring environmental triggers or large spaces. Additionally, larval rearing demands precise and continuous osmoregulatory management. Fluctuations in water salinity outside the narrow optimal range found in this study (10 g/L) can rapidly induce high larval mortality or inhibit egg hatching, leading to colony collapse. Overcoming these ecological barriers to maintain a viable line up to the F17 generation provides a unique and highly valuable biological tool for operational vector control research in West Africa.

Insecticide resistance tests revealed confirmed resistance to alpha-cypermethrin (81.7%) and suspected resistance to deltamethrin and permethrin, thereby highlighting a loss of pyrethroid efficacy in this area. The full restoration of susceptibility following PBO pre-exposure demonstrates the predominant role of cytochrome P450 enzymes. This finding is consistent with recent genomic analyses; indeed, Whole Genome Sequencing (WGS) studies conducted on *An. melas* populations in West Africa have revealed duplications of the CYP9K1 gene, a major marker of metabolic pyrethroid resistance within the *An. gambiae* complex [14]. Furthermore, our results corroborate the work of Sy et al. (2021) [5], in Senegal, who had already identified kdr mutations in this species, suggesting the coexistence of target-site modification and enzymatic detoxification resistance mechanisms [15]. The observation of persistent resistance at the 5× dose for alpha-cypermethrin (95.5%) followed by a full restoration of susceptibility at 10× (99.1%) classifies this population within a moderate resistance intensity category according to standard complex-wide benchmarks. This situation is concerning as it exceeds the efficacy thresholds of standard insecticide-treated nets. However, the efficacy restored by PBO confirms that PBO-LLINs (next-generation nets) constitute a robust alternative. These results align with the regional trend observed in The Gambia and Guinea-Bissau, where the efficacy of conventional interventions is increasingly challenged by the exophilic behaviour and physiological plasticity of *An. melas* [3,4,5,6,7,8,9,10,11,12,13,14,15,16].

The salinity recorded in Mbine Coly (10–18 g/L) confirms the halophilic specialisation of *An. melas*. This tolerance to brackish environments provides it with a selective advantage over *An. arabiensis* in mangrove areas [2]. This environmental factor, coupled with its ability to bite outdoors (exophagy), explains why this species ensures residual transmission where other vectors are controlled by IRS or traditional LLINs [6]. Nevertheless, the full susceptibility observed to pirimiphos-methyl and bendiocarb offers serious avenues for insecticide rotation, particularly through indoor residual spraying.

A potential limitation of this study is the relatively small number of founding individuals used to establish the laboratory colony. Initiating the strain with 26 females may have induced a genetic bottleneck, a common phenomenon in mosquito colonization that can restrict genetic diversity and accelerate genetic drift. This restriction might influence the frequency of resistance alleles, potentially causing variations between our colonized strain and the original wild population. Nevertheless, considering that An. melas is an eco-physiologically sensitive species that is exceptionally challenging to colonize, achieving a stable F17 line is a significant step forward. Future molecular monitoring, including the sequencing of targeted metabolic genes such as CYP9K1, will be essential to evaluate allelic stability and better characterize the micro-evolutionary dynamics of this colony over time.

## 5. Conclusions

This study documents, for the first time, the multi-insecticide susceptibility profile of an *Anopheles melas* population in Mbine Coly (Mbour), while marking a significant technical advancement in maintaining this species under insectarium conditions, now reaching the F17 generation. The stabilization of this wild strain into a sustainable colony provides a valuable biological tool for research. Our findings confirm that the *Anopheles melas* population from Mbine Coly (Mbour) has developed significant resistance to pyrethroids, driven by metabolic mechanisms that can be neutralized by PBO.

These results necessitate immediate strategic actions, such as the priority deployment of next-generation Long-Lasting Insecticidal Nets (PBO-LLINs) in the Mbour district. Furthermore, insecticide rotation in IRS programmes, involving the use of carbamates or organophosphates, should be considered for insecticide resistance management. Additionally, molecular characterization of metabolic resistance genes will be necessary to confirm the specific enzymatic pathways driving this resistance in West African *An. melas* populations.

## Figures and Tables

**Table 1:** Comparative hatching rates, development times, and cumulative larval survival across the tested salinity gradients (0 g/L, 10 g/L, and 18 g/L).

Salinity Level (g/L)	Mean Hatching Time (h)	Larval Development Time (Days)	Cumulative Larval Survival Rate (%)	*p*-Value (vs. 10 g/L)
0 g/L(Freshwater)	38.4 ± 4.2	11.2 ± 1.5	71.0%	<0.01
10 g/L (Optimal)	24.2 ± 2.1	8.4 ± 0.8	86.5%	—
18 g/L (High Salinity)	31.8 ± 3.6	9.9 ± 1.2	74.2%	0.024

**Table 2:** Susceptibility status of the *An. melas* population from Mbine Coly (Mbour) to pyrethroids.

District	Salinity (g/L)	Molecule	Alive	Dead	Total Tested	Mortality (%)
Mbour	10/18	Deltamethrin (0.05%) 1×	9	95	104	91.3
		Deltamethrin 0.05% after PBO	0	110	110	100
		Deltamethrin 5× (0.25%)	0	110	110	100
		Permethrin (0.75%) 1×	6	100	106	94.3
		Permethrin 0.75% after PBO	0	112	112	100
		Permethrin 5× (3.75%)	0	110	110	100
		Alpha-cypermethrin (0.05%) 1×	19	85	104	81.7
		Alpha-cypermethrin 0.05% after PBO	4	105	109	96.3
		Alpha-cypermethrin 5× (0.25%)	5	107	112	95.5
		Alpha-cypermethrin 10× (0.5%)	1	116	117	99.1

**Table 3:** Observed mortality after exposure of the Mbine Coly strain to organophosphates and carbamates.

District	Salinity (g/L)	Molecule	Alive	Dead	Total Tested	Mortality (%)
Mbour	10/18	Pirimiphos-methyl (0.25%)	0	110	110	100
		Bendiocarb (0.1%)	0	110	110	100

## Data Availability

The data presented in this study are available on request from the corresponding authors.

## Abbreviations

CDC	Centers for Disease Control and Prevention
IRS	Indoor Residual Spraying
kdr	Knockdown resistance
LLINs	Long-Lasting Insecticidal Nets
MARCAD	Malaria Research Capacity Development
PBO	Piperonyl Butoxide
RH	Relative Humidity
WGS	Whole Genome Sequencing
WHO	World Health Organization
